# Effects of whole-body vibration warm-up on subsequent jumping and running performance

**DOI:** 10.1038/s41598-023-34707-6

**Published:** 2023-05-07

**Authors:** Paulina Ewertowska, Katarzyna Świtała, Wojciech Grzyb, Robert Urbański, Piotr Aschenbrenner, Michał Krzysztofik

**Affiliations:** 1 Chair of Health and Biological Sciences, Gdansk University of Physical Education and Sport, Gdańsk, Poland; 2 Faculty of Physical Education, Gdansk University of Physical Education and Sport, Gdańsk, Poland; 3 Department of Biomechanics and Sports Engineering, Gdansk University of Physical Education and Sport, Gdańsk, Poland; 4 Institute of Sport Sciences, The Jerzy Kukuczka Academy of Physical Education in Katowice, Mikołowska 72A Str., 40-065 Katowice, Poland; 5 Department of Sport Games, Faculty of Physical Education and Sport, Charles University in Prague, Prague, Czech Republic

**Keywords:** Health care, Medical research

## Abstract

The aim of this study was to examine whether acute whole-body vibration, a single bout of drop jumps, or a combination of both may enhance countermovement jump (CMJ) and would affect volitional pace 3 km running performance. Twelve healthy and recreationally active males completed 4 conditions in randomized order: (i) 5 sets of 30 s calf raises on the platform but without vibration; (ii) 5 sets of 30 s calf raises on the vibration platform with 30 s rest intervals between sets; (iii) 5 sets of 6 drop jump with a 30 s rest interval between sets; (iv) 5 sets of 30 s calf raises on the vibration platform followed by 6 drop jumps with a 30 s rest interval between sets. Before, 3-min after, and immediately after a 3 km run each participant performed CMJ. No significant difference between conditions (p = 0.327) for the 3 km time trial was found. Whereas CMJ height and relative peak power were significantly improved in post-3 km run than at baseline (p < 0.001 and p = 0.025) and post-warm-up (p = 0.001 and p = 0.002) in all conditions. The present study indicates that warm-up consisting of either whole-body vibration, drop jumps, or a combination of both failed to acutely improve CMJ and 3 km volitional pace running performance in physically active males. However, the increase in the CMJ performance was noted after the end of the 3 km run, which may indicate that the warm-up protocols used were insufficient to enhance subsequent performance.

## Introduction

A warm-up is performed prior to exercise to prevent possible injury and enhance subsequent performance. An increase in muscle temperature, blood flow, and improved readiness of the neuromuscular system^1–3^ are the main mechanisms underpinning the positive effects of warming up. Research shows that a warm-up as short as 5 min of running can significantly improve the efficiency of explosive tasks. For example, Young and Behm^4^ found that a 4 min run at a volitional pace enhanced CMJ performance. Consistent with this Andrade^5^ found that a 5 min run at 70% of maximum heart rate augmented CMJ performance. In addition, Vetter^6^ reported no differences in CMJ height after a walk and a running warm-up (4 min walk and 2 min run at volitional pace) in comparison to the same, yet extended warm-up by sets of several exercises (i.e., toe raises, high knees lift marching, jump), as well as extended by dynamic stretching (i.e., quadriceps and Achilles stretches). Therefore, there is a minimum effective warm-up duration needed to achieve the desired effect, while the extension of which may not bring any additional performance improvement or may even worsen the subsequent activity.

Despite the undeniable benefits of warm-up, methods are still being sought that will further increase its effectiveness, but also shorten its duration and reduce the associated effort^1,2,7^. Since, temperature-related mechanisms have been suggested as a main explanation for the positive effects of warm-up, passive warm-up techniques including heat garments, hot water immersion, and saunas which allow for a rapid and significant increase in muscle temperature without depleting energy substrates, have been proposed^1^. On the other hand, the whole-body vibration modality has been considered as a warm-up or as a part of the warm-up, which might provide similar or additional performance improvement with a lower effort required in comparison to traditional warm-up protocols^8–11^. In the case of whole-body vibration warm-up, possibly the neuromuscular stimulation via tonic vibration reflex^8^ is a purported mechanism underpinning changes in explosive performance instead of muscle temperature increases.

Cardinale and Lim^12^ reported a squat jump height increase after 5 min of whole-body vibration (semi-squatting position with vibration frequency at 20 Hz and peak-to-peak displacement of 4 mm) in recreationally trained participants (2 females and 13 males). In accordance, Cormie et al.^8^ showed an increase in CMJ height immediately after a single bout of 30 s whole-body vibration (half-squatting position with vibration frequency at 30 Hz and peak-to-peak displacement of 2.5 mm) in recreationally trained males. Similarly, a recent study by Wu et al.^11^ indicated an enhancement in the maximum rate of force development during CMJ performed 1 and 2 min after a single bout of 60 s whole-body vibration (half-squatting position with vibration frequency at 30 Hz and peak-to-peak displacement of 2 mm) in elite male volleyball players. Although, whole-body vibration seems to be a promising modality to acutely improve explosive performance far less is known about the impact of acute exposure to vibration within the context of endurance performance. A study by Padulo et al.^13^ found that ten bouts of 60 s whole-body vibrations (half-squatting with raised heels with vibration frequency at 45 Hz and peak-to-peak displacement of 2.2 mm) affected running gait during a 5 min run in male marathon runners. It seems, therefore, that the vibration stimulus may have a different effect on short-term explosive tasks and prolonged endurance tasks. It's interesting to note that only squat exercise modifications during whole-body vibration were utilized in the studies mentioned above^8,11–13^. However, in addition to the upper leg muscle, the ankle plantar flexor muscles play a major role in producing propulsive force and have a crucial impact on running and jumping performance^14–16^. Therefore, it would be interesting to examine the effect of exercises involving the ankle muscles on subsequent running and jumping performance from the perspective of exercise selection during whole-body vibration conditioning.

Considering warm-up procedures, individuals may also try to exploit the effect of post-activation performance enhancement to further the benefits following a warm-up. An example would be an expected increase in performance (i.e. in vertical jumping) a few minutes after performing a low-volume conditioning maximal voluntary contraction like a plyometric exercise (i.e. a drop jumps at the end of the warm-up)^17^. There is considerable literature supporting the positive benefits of post-activation performance enhancement in explosive tasks, but current evidence suggests that endurance activities can also benefit from conditioning exercises implemented in warm-up routines^18–20^. There are two studies that showed improved running performance after the inclusion of a single bout of drop jumps into the pre-running warm-up^18,21^. Blagrove et al.^21^ observed improvement in running economy during a run to exhaustion following a set of six drop jumps among well-trained young runners. Furthermore, Boullosa et al.^18^ reported a faster 1000 m run time after a set of five drop jumps compared to a control condition (without drop jumps) in elite male endurance runners. However, to the best of the author's knowledge, there is a lack of studies that examined the acute impact of whole-body vibration or in a combination with plyometric exercises (like drop jumps) on endurance running performance. Moreover, there is little conclusive evidence regarding acute WBV protocols in comparison to very low volume and intensity exercises, a combination of both, and finally, whether these are comparable or even superior to a simple running warm-up in enhancing subsequent athletic performance.

The objective of this study was twofold. First, to examine whether acute whole-body vibration, a single bout of drop jumps, or a combination of both may enhance CMJ performance. Second, we attempted to evaluate if (i) performed warm-up protocols would affect volitional pace running and (ii) if this running would have an additional effect on jumping ability above and beyond that of the applied warm-up procedures. Given that previous studies showed enhancement in explosive performance after whole-body vibration^8–11^ we hypothesized that they will be also effective in the current study by improving CMJ performance without impacting running performance. Moreover, we assumed no additional benefits of running on subsequent CMJ.

## Materials and methods

### Experimental approach to the problem

**Figure 1:**
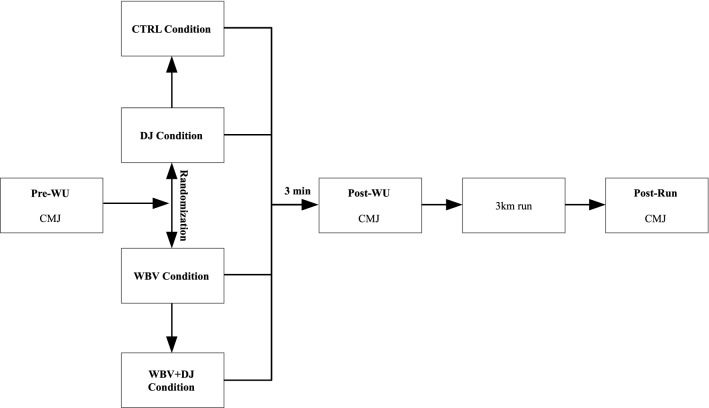
Study design flowchart. *WU* warm-up, *CMJ* countermovement jump, *DJ* drop jump condition, *CTRL* control condition, *WBV* whole body vibration condition, *WBV* + *DJ* whole body vibration and drop jump condition.

The participants took part in four experimental sessions within 4 weeks. The experimental sessions were performed in a randomized order, 1 week apart, where each participant performed: (i) 5 sets of 30 s calf raises on the platform but without vibration (CTRL); (ii) 5 sets of 30 s calf raises on the vibration platform with 30 s rest intervals between sets (WBV); (iii) 5 sets of 6 drop jump with a 30 s rest interval between sets (DJ); iv) 5 sets of 30 s calf raises on the vibration platform followed by 6 drop jumps with a 30 s rest interval between sets (WBV + DJ). To examine the acute effects of each conditioning protocol on subsequent countermovement jump height a single-sets of 2 repetitions of the CMJ were performed before, 3-min after, and immediately after a 3 km run (Fig. 1).

### Subjects

Twelve healthy and recreationally active males^22^ (age: 38 ± 4 yrs; body mass: 76.2 ± 9.8 kg; height: 179 ± 8 cm; regular aerobic and resistance training of approximately 60 min 2 times per week) participated in the study. The participants had at least 5 years of running training experience and were familiar with running on the treadmill. Furthermore, they were free from any musculoskeletal injuries 6 months prior to enrollment into the study. Participants were asked to maintain their normal dietary and sleep habits throughout the study, not to use any supplements or stimulants, and not to perform any strenuous effort 24-h prior to testing. The study participants were allowed to withdraw from the experiment at any moment and were free from musculoskeletal disorders. They were informed about the benefits and potential risks of the study before providing their written informed consent for participation but were not informed about the expected results. The study protocol was approved by the Bioethics Commission at the District Medical Chamber in Gdansk, Poland (KB–57/22), and performed according to the ethical standards of the Declaration of Helsinki, 2013. The sample size was similar to previous studies of whole-body vibration on jumping performance^23,24^. Considering the applied 2-way analysis of variance (ANOVA) (4 conditions and 3 repeated measures), 12 participants, an alpha-error < 0.05, and correlation among repeated measures = 0.5, an effect size of at least 0.4 is needed to get the power above 0.8 (G*Power, Dusseldorf, Germany).

### Procedures

#### Experimental sessions

In a randomized and counterbalanced order, the participants performed 4 different testing conditions, one week apart: (i) 5 sets of 30 s calf raises on the platform but without vibration (CTRL); (ii) 5 sets of 30 s calf raises on the vibration platform with 30 s rest interval between sets (WBV); (iii) set of 6 drop jumps (DJ); (iv) 5 sets of 30 s calf raises on the vibration platform with a 30 s rest interval between sets followed by a set of 6 drop jumps (WBV + DJ). To assess changes in jumping variables, single sets of 2 repetitions of the CMJ were performed before and 3 min after the conditioning activity as well as immediately after a 3 km run.

The whole-body vibration was performed on a commercial machine (LADY 1 Pro, New Life Balance GMBH production, Germany). Briefly, the device provides a rotational type of vibration and is 50 cm in width and 25 cm in height above the floor. The vibration frequency and peak-to-peak displacement were respectively set at 20 Hz and 5 mm, and it was constant throughout the intervention. The device was placed on the ground, and a handrail was mounted to facilitate balance control during interventions. The participant stood parallel barefoot, on midfoot in a semi-squat position with their feet hip-width distance apart^25,26^. During the CTRL, WBV, and WBV + DJ conditions, in order to perform a calf raise exercise, the participants were instructed to "raise heel as far up onto their toes as possible" with a constant movement tempo of 1 s for both concentric and eccentric phases. The protocol included 5 sets of 30 s vibration with 30 s rest intervals (total exposure 2 min 30 s). The intervention was supervised by two experienced trainers.

The drop jumps were performed from a 51-cm high box. The participant started in the standing position with hands placed on the hips. To initiate the drop action, the participants were instructed to: “step off” the box one foot at a time and “jump up as fast as possible after contact with the ground, making sure that the jump is the highest possible”. The jumps were interspersed by approximately 15-s intervals.

The selection of warm-up variables was based on previous studies that showed their effectiveness in inducing performance enhancement^12,27^. Specifically, a conditioning protocol using a single set of drop jumps found an acute enhancement of jumping and running performance^18,20,27,28^. In addition, the vibration variables were selected based on a previous study that showed effectiveness in acute jumping enhancement^12^. Finally, since the upper leg and ankle muscles play a major role in producing a propulsive force that accelerates an athlete upwards and forwards^14,29–31^ we decided to select exercises that will highly engage those muscles during warm-up.

#### Measurement of countermovement jump performance

Countermovement jump performance was measured using force plates (9286BA, Kistler Instruments AG, Winterthur, Switzerland). The ground reaction force was recorded with a sampling frequency of 1000 Hz and was used to calculate take-off vertical velocity according to the impulse-momentum theorem. This device has been previously confirmed as valid and reliable^32^ for assessing vertical jump kinematics. Each participant performed two CMJ without arm swings before conditioning as a baseline, 3 min postconditioning, and immediately after a 3 km run. For this measurement, the participant started in the standing position with hands placed on the hips. Next, they dropped into the countermovement position to a self-selected depth, followed by a maximal effort vertical jump. The participants were instructed to jump as high as possible and land in the same position as the take-off in the mid-section of the force plate. After each jump, the participant reset to the starting position, and the procedure was completed for a total of two jumps. Jump height (JH), relative peak power (RPP), and contraction time (CT) were evaluated. The best jump in terms of height was retained for further analysis. The jump height was calculated from the vertical velocity of the center of mass at take-off using the equation:

$$Jump \; height=\frac{{TOV}^{2}}{2g},$$

where: TOV—vertical velocity of the center of mass at take-off; $g=9.81 \mathrm{m} \; {\mathrm{s}}^{-2}$

#### Volitional pace 3 km running performance

The 3 km time trial was performed on a conventional motorized treadmill with an incline set at 0% (Saturn, HP Cosmos, Nussdorf Traunstein, Germany). Participants were blinded to their running speed and elapsed time, and the researcher provided standard encouragement every 500 m. The participants were also partially blinded to the distance traveled; however, the last 500 m were announced. Participants were instructed to tell the researcher whether they wanted the speed to be increased or decreased at any point during the test by saying "faster" or "slower." Furthermore, the participants were instructed to finish the run as quickly as possible, as they would in a competitive event.

### Statistical analysis

A Kolmogorov–Smirnov test was used to test the normal distribution of the data, and Mauchly's test was used to test for the assumption of sphericity. A single-rater intra-class correlation coefficient (ICC) and coefficient of variability (CV) were used to measure the analyzed variables' reliability (calculated from the baseline measurements taken for each of the dependent variables)^33^.

One-way repeated measures ANOVA was used to examine differences in 3 km running time. Two-way repeated measures ANOVAs (4 × conditions [CTRL; WBV; DJ; WBV + DJ] × 3 time-points [pre-WU; post-WU; post-RUN]) were used to investigate the influence of warm-up type and 3 km run on jumping ability variables. When a significant interaction or main effect was found, post hoc tests with Bonferroni correction were used to analyze the pairwise comparisons. The magnitude of mean differences was expressed with standardized effect sizes. Thresholds for qualitative descriptors of Hedges g were interpreted as ≤ 0.20 “small”, 0.21–0.79 “medium”, and > 0.80 as “large”^34^.

All data were analyzed using SPSS (version 25.0; SPSS, Inc., Chicago, IL, USA) and are reported as the means with standard deviations and their 95% confidence intervals (CIs). Statistical significance was set at p < 0.05.

## Results

The Kolmogorov–Smirnov tests did not indicate any violation of data distribution for CMJ variables. The ICC and CV results are presented in Table 1.

**Table 1:** Intersession reliability of the analyzed variables.

Variable	ICC (95%CI)	CV (SD)
Jump height	0.916 (0.801–0.973)	5.8% (4.5%)
Relative peak power	0.786 (0.477–0.931)	7.4% (6.2%)
Contraction time	0.843 (0.623–0.949)	10.3% (5.6%)

*ICC* intraclass correlation coefficient, *CV* coefficient of variation, *SD* standard deviation.

A one-way ANOVA didn’t show a significant difference between conditions (F = 1.193; p = 0.327) for 3 km time trial (Table 2).

**Table 2:** Results of the 3 km time trial.

	CTRL	WBV	DJ	WBV + DJ
Time [mm:ss]	15:03 ± 00:50	14:48 ± 1:35	14:52 ± 1:13	14:29 ± 1:20

*CTRL* control condition, *DJ* drop jump condition, *WBV* whole body vibration condition, *WBV* + *DJ* whole body vibration and drop jump condition.

### Countermovement jump performance

**Figure 2:**
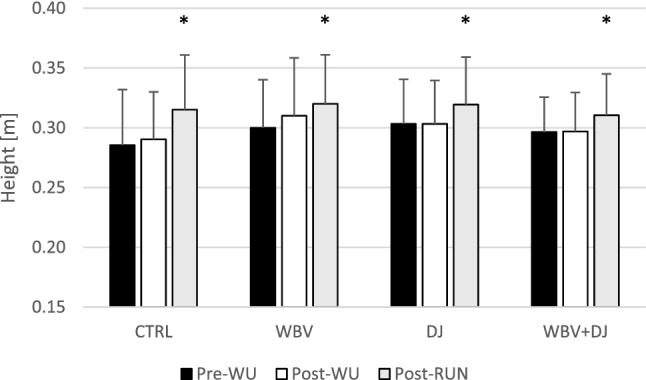
Jump height values during countermovement jump performance at subsequent time points.

A two-way ANOVA did not show a significant interaction (F = 0.815; p = 0.562) but a significant main effect of condition (F = 3.058; p = 0.042) and time (F = 24.041; p < 0.001) for jump height was found. The post-hoc comparisons for the main effect of the condition indicated significantly higher CMJ heights during the WBV condition in comparison to the CTRL condition (p = 0.043; ES = 0.31). Whereas the post-hoc comparisons for the main effect of time showed that significantly higher CMJ heights were reached in post-RUN compared to pre-WU (p < 0.001; ES = 0.53) and post-WU (p = 0.001; ES = 0.42) (Fig. 2).

**Figure 3:**
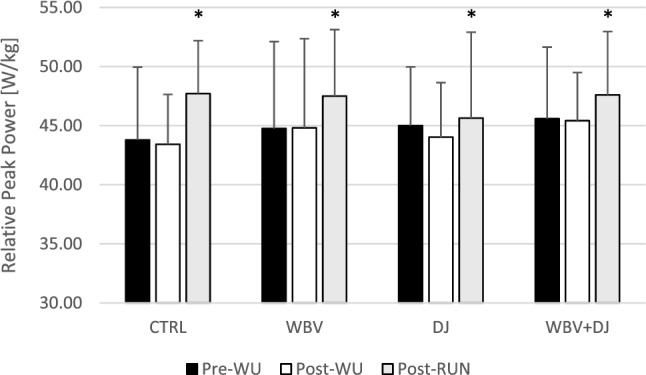
Relative peak power values during countermovement jump performance at subsequent time points.

A two-way ANOVA did not show a significant interaction (F = 0.703; p = 0.55) or main effect of condition (F = 0.762; p = 0.523) but a significant main effect of time (F = 9.249; p = 0.001) for relative peak power was found. The post-hoc comparisons for the main effect of time showed significantly greater RPP in post-RUN compared to pre-WU (p = 0.025; ES = 0.39) and post-C (p = 0.002; ES = 0.48) (Fig. 3).

**Figure 4:**
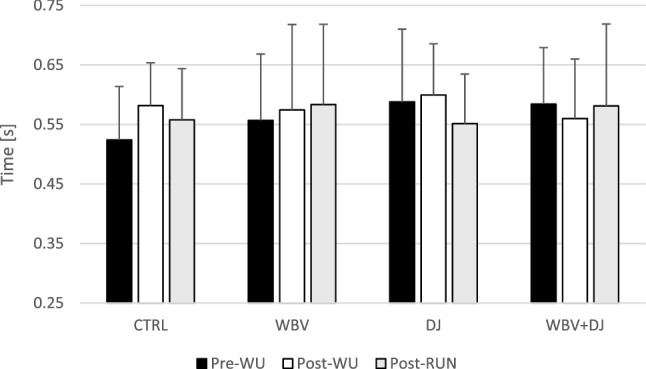
Contraction time values during countermovement jump performance at subsequent time points.

A two-way ANOVA did not show a significant interaction (F = 1.408; p = 0.225) or main effect of condition (F = 0.451; p = 0.718) and time (F = 0.465; p = 0.634) for contraction time (Fig. 4).

## Discussion

The main purpose of this study was to determine whether whole-body vibration, drop jumping, or a combination of both can contribute to the acute improvement of CMJ performance. Additionally, a 3 km volitional pace running was then performed, followed by CMJ, to examine if continuing the warm-up would have an additional effect on jumping ability. The main finding of this experiment was that none of the warm-ups applied had an effect on both jumping and running. In turn, the 3 km volitional pace running significantly improved the CMJ performance for each of the conditions.

Surprisingly, the results of this study contradict some reports regarding the acute effect of whole-body vibration^8,10–12^, a combination of whole-body vibration and drop jumps^23^, as well as a single bout of drop jumps only^27^ on CMJ performance. For example, studies by Cardinale and Lim and Cormie et al. showed an acute improvement in vertical jumps height after the whole-body vibration, despite extremely different exposure times (5 min vs. 30 s) and similar vibration parameters (vibration frequency at 20 Hz and peak-to-peak displacement of 4 mm vs. vibration frequency at 30 Hz and peak-to-peak displacement of 2.5 mm). However, in both studies, the participants were in a semi-squat position during the vibration, while in the current study, they additionally performed calf raises. Therefore, it is possible that the vibration stimulus used in the current study was ineffective because it induced a level of fatigue that suppressed the positive effects of the warm-up, and that the 3-min interval after the vibration stimulus was too short and fatigue still hadn't subsided. Nevertheless, this is not the first study finding no effects of whole-body vibration on explosive performance^35,36^. For instance, Torvinen et al.^35^ reported an insignificant increase in vertical jump height (+ 1.6%) immediately after 4 min of light exercises (i.e., squatting, standing, light jumping) performed during whole-body vibration (with increasing vibration frequency by 5 Hz, from 25 Hz for the first min until 40 Hz for the last min and amplitude of 2 mm) in physically active females and males. Similarly, a recent study by Feland et al.^36^ found no significant increase in CMJ performed 2 min following 10 min of whole body vibration (half squatting position with a vibration frequency of 26 Hz and amplitude of 3.6 mm) among college-aged students. The various findings could be the outcome of differences in vibration variables being employed. Compared to studies in which similar vibration stimulus variables were used and their positive effect on CMJ performance was demonstrated (frequency 20–30 Hz, amplitude 2–4 mm)^8,11,12^, in a study by Feland et al.^36^, a much longer duration of vibration (10 min) was applied. Torvinen et al.^35^ used a slightly higher frequency (35 and 40 Hz for 2 min) and participants performed exercises during the vibration stimulus. Although the exercises were considered very light and did not cause fatigue, concomitant surface electromyographic analysis of the vastus lateralis and gluteus medius muscles indicated a decrease in mean power frequency during vibration, which indicated evolving fatigue^36^.

In addition, a single bout of drop jumps was ineffective in the acute improvement of CMJ performance, despite previous studies finding significant enhancement of jumping performance following a set of low volume drop jumps (5–6 repetitions)^20,27,37^. Likewise, the combination of whole-body vibration followed by drop jumps had no impact on subsequent CMJ performance in the current study, while a recent study by Dallas et al.^23^ found this conditioning to be effective. It may appear that the characteristics of the participants in the current study were related to no effects of whole-body vibration, drop jumps, or a combination of the two on CMJ performance. Wu et al.^11^ and Chen et al.^27^ studied male volleyball players, in a study by Dallas et al.^23^ competitive gymnasts participated, while Cormie et al.^8^ examined males involved in resistance training, and Cardinale and Lim^12^ tested physically active females and males. Indeed, participants of the current study were also physically active, however, without experience in resistance training. This in part has been confirmed by Cloak et al.^38^ who revealed an increase in peak knee isometric force following 3 sets of 60 s of whole-body vibration at 40 Hz and 8 mm peak-to-peak amplitude among professionals but not in amateur soccer players. Moreover, also in the case of the post-activation performance enhancement effect which might occur due to the use of drop jumps or their combination with whole-body vibration as previously indicated^23^, the participants' characteristics could have influenced the findings. Strength level, gender, training background, and experience have been found to be impactful moderators of the magnitude of performance improvement due to the post-activation performance enhancement effect^39–41^. For example, a larger performance enhancement has been reported among stronger (≥ 1.75 back squat/body mass ratio and ≥ 1.35 bench press/body mass ratio) and experienced in resistance training (> 2 years) than in weaker individuals^40^. Therefore, further studies should clarify these issues by comparing different populations considering mentioned moderators under whole-body vibration procedures.

Moreover, to the best of the author's knowledge, there is a lack of studies that examined the acute impact of whole-body vibration or in a combination with drop jumps on endurance running performance. A study by Padulo et al.^13^ indicated a negative effect of whole-body vibration on running gait in marathon runners. In regards to the inclusion of drop jumps in the pre-running warm-up, Boullosa et al.^18^ showed an improvement in 1000 m running time after 5 drop jumps among male elite distance runners. However, in the 3 km volitional run assessed in the current study, no significant differences in time were found. Due to the lower training level of participants in the current study than those in studies by Padulo et al.^13^ and Boullosa et al.^18^, or simply because warm-ups used were insufficient to induce meaningful post-activation performance enhancement effect.

Interestingly, regardless of the warm-up type, improvements in CMJ performance were only noted after the 3 km volitional run (lasting approximately 15 min). Therefore, this might simply mean that the warm-up protocols used were insufficient in terms of volume or that bodyweight exercises may not provide beneficial effects in this population. Considering, the post-activation performance enhancement effect, in the recent metanalysis Brink et al.^42^ showed that using bodyweight conditioning activities may acutely improve the performance outcome of a subsequent task. On the other hand, a greater conditioning activity volume is recommended for weaker individuals than for stronger ones^40^. This may be an explanation why in the study by Boullosa et al.^18^, elite endurance runners improved CMJ at 3 min after 5 drop jumps with no further improvement after a 1000 m run, whereas in the current study no difference in CMJ after drop jumps were noted, but they were enhanced after the 3 km volitional run. However, to the best of the authors’ knowledge, there is no data available comparing different whole-body vibration durations in regard to the participant's fitness level. Nevertheless, fast-twitch fibers are possibly more sensitive to vibration^43^ and individuals with a higher proportion of type II muscle fibers derive a greater post-activation performance enhancement benefit^44^. Therefore, it is plausible that the lack of improvements following the examined warm-ups was related to their short duration and low fitness level of participants in the current study. However, it cannot be ruled out that if the measurements would be made also at later time points after conditioning, the performance outcomes could differ.

Certain limitations of the current study have to be mentioned. First, we assessed only a single time point after the applied warm-ups (3 min), so there is no certainty that any change could be noted at another time point. Secondly, we only measured the duration of the run without evaluating the running gait kinematics, and we did not assess subjective emotions related to the effort made. Furthermore, we did not measure whether the tonic vibration reflex was evoked.

## Conclusions

The results of the present study indicate that warm-up consisting of either whole-body vibration, drop jumps, or a combination of both failed to acutely improve countermovement jump and 3 km volitional pace running performance in physically active males. Interestingly, the increase in the countermovement jump height was noted after the end of the 3 km volitional run, which may indicate that the warm-up protocols used were insufficient to enhance subsequent performance. Therefore, based on the current findings, the use of whole-body vibration as a part of warm-up procedures should be carefully considered, and keep in mind that this approach may require additional activities to reach optimal warm-up stimuli.

## Data Availability

The datasets used and/or analysed during the current study available from the corresponding author on reasonable request.
